# P-1358. Understanding Fungal Infections and Trends in Fungal Resistance: A Single Center Evaluation

**DOI:** 10.1093/ofid/ofae631.1535

**Published:** 2025-01-29

**Authors:** Nuzhat Islam, Nina Haste, Ahnika Kline, Shira Abeles

**Affiliations:** UC San Diego Health, San Diego, California; University of California San Diego, San Diego, California; UC San Diego, San Diego, California; University of California San Diego, San Diego, California

## Abstract

**Background:**

Invasive fungal infections cost the US health care system billions and are challenging to treat. In 2021 alone, the CDC estimates that fungal infections contributed to over $7 billion in direct medical costs and over 7,000 deaths in the US. The most common agents used to treat clinical fungal infections are azoles. However, antifungal resistance is on the rise. Exposure to agricultural fungicides has been identified as likely contributing to increased resistance to medical azoles, as there has been incidence of infection with azole-resistant fungi in patients who were azole-naïve. Furthermore, *in vitro* studies have demonstrated that fungi exposed to agricultural azoles develop cross-resistance to medical azoles. This study aims to describe retrospective antifungal susceptibility trends for medical azoles in patients at UCSD Health and agricultural azole use in San Diego and Imperial Counties.

**Methods:**

Chart review was used to obtain sensitivity data (minimum inhibitory concentrations or MICs) for non-*Candida* fungal isolates sent for susceptibility testing from UCSD Health patients from January 2016 through February 2024. Data on fungal isolates including species, date of isolate collection, susceptibility data, and patient zip code were collected. Agricultural azole use data is publicly available from the California Department of Pesticide Regulation and was analyzed for azoles applied in San Diego and Imperial Counties from 2016 to 2021.

**Results:**

189 fungal isolates from 170 patients were included in the analysis. *Fusarium* demonstrated high resistance to common medical azoles which did not change over time. *A. fumigatus* also exhibited minimal change in MICs for itraconazole and posaconazole but showed possible increase in MICs over time for voriconazole and isavuconazole. Imperial County had an 8 to 10-fold higher use of agricultural azoles compared to San Diego County.

**Conclusion:**

*Fusarium* demonstrated resistance to all common medical azoles. There may be a possible increase in resistance of *A. fumigatus* to voriconazole and isavuconazole over time, however this is limited by small sample size. This study highlights the need for increased antifungal susceptibility testing at UCSD Health.

**Disclosures:**

**Nina Haste, PharmD, PhD**, Novartis: Spouse is an employee

